# Icosapent ethyl therapy for very high triglyceride levels: a 12-week, multi-center, placebo-controlled, randomized, double-blinded, phase III clinical trial in China

**DOI:** 10.1186/s12944-023-01838-8

**Published:** 2023-06-10

**Authors:** Zhen Wang, Xin Zhang, Yanling Qu, Shuyang Zhang, Yundai Chen, Xiaoping Chen, Xin Qi, Peijing Liu, Shuqin Liu, Shan Jiang, Ronghai Man, Liping He, Ling Wu, Zhanquan Li, Yijun Shang, Zhaohui Qiu, Feng Liu, Chenhong Xu, Chunlin Lai, Junbo Ge

**Affiliations:** 1 Department of Cardiology, Shanghai Institute of Cardiovascular Diseases, Zhongshan Hospital, Fudan University, Shanghai, China; 2 Department of Cardiac Function, The First Affiliated Hospital of Baotou Medical College, Inner Mongolia University of Science and Technology, Baotou, Inner Mongolia China; 3 Department of Cardiology, Yuncheng Central Hospital, Yuncheng, Shanxi China; 4 Department of Cardiology, Peking Union Medical College Hospital, Peking Union Medical College & Chinese Academy of Medical Sciences, Beijing, China; 5 Department of Cardiology, Chinese PLA General Hospital, Beijing, China; 6 Department of Cardiology, West China Hospital, Sichuan University, Chengdu, China; 7 Department of Cardiology, Tianjin Union Medical Center, Tianjin, China; 8 Department of Cardiology, Affiliated Hospital of Jiangsu University, Zhenjiang, Jiangsu China; 9 Department of Cardiology, Liaocheng People’s Hospital and Liaocheng Clinical School of Taishan Medical University, Liaocheng, Shandong China; 10 Department of Cardiology, Jinzhou Central Hospital, Jinzhou, Liaoning China; 11 Department of Cardiology, Chifeng Municipal Hospital, Chifeng, Inner Mongolia China; 12 Department of Cardiology, Inner Mongolia People’s Hospital, Huhhot, China; 13 Department of Cardiology, General Hospital of Ningxia Medical University, Yinchuan, China; 14 Department of Cardiology, Liaoning Provincial People’s Hospital, Shenyang, China; 15 Department of Cardiology, Jilin Central General Hospital, Jilin, China; 16 Department of Cardiology, Shanghai Tongren Hospital, Shanghai Jiao Tong University School of Medicine, Shanghai, China; 17 Departments of Cardiology, Suzhou Kowloon Hospital, School of Medicine Shanghai Jiaotong University, Suzhou, Jiangsu China; 18 Department of Cardiology, Jingzhou Central Hospital, Jingzhou, Hubei China; 19 Department of Cardiology, Shanxi Provincial People’s Hospital, Taiyuan, China

**Keywords:** Icosapent ethyl, Hypertriglyceridemia, Triglyceride, Low-density lipoprotein cholesterol

## Abstract

**Objectives:**

Eicosapentaenoic acid in its ethyl ester form is the single active component of icosapent ethyl (IPE). This study was a phase III, multi-center trial assessing the safety and efficiency of IPE for treating very high triglyceride (TG) in a Chinese cohort.

**Methods:**

Patients having TG levels (5.6–22.6 mmol/L) were enrolled and randomly assigned to receive a treatment of oral intake of 4 g or 2 g/day of IPE, or placebo. Before and after 12 weeks of treatment, TG levels were assessed and the median was calculated to determine the change between the baseline and week 12. In addition to examining TG levels, the impact of such treatments on other lipid changes was also investigated. The official Drug Clinical Trial Information Management Platform has registered this study (CTR20170362).

**Results:**

Random assignments were performed on 373 patients (mean age 48.9 years; 75.1% male). IPE (4 g/day) lowered TG levels by an average of 28.4% from baseline and by an average of 19.9% after correction for placebo (95% CI: 29.8%-10.0%, *P* < 0.001). In addition, plasma concentration of non-high-density lipoprotein cholesterol (non-HDL-C), very low-density lipoprotein (VLDL) cholesterol, and VLDL-TG remarkedly reduced after IPE (4 g/day) treatment by a median of 14.6%, 27.9%, and 25.2%, respectively compared with participants in placebo group. Compared to the placebo, neither 4 nor 2 g of IPE daily elevated LDL-C levels with statistical significance. IPE was well tolerated by all the treatment groups.

**Conclusions:**

IPE at 4 g/day dramatically lowered other atherogenic lipids without a noticeable increase in LDL-C, thereby decreasing TG levels in an exceptionally high-TG Chinese population.

## Introduction

Atherosclerotic cardiovascular disease, also referred to as ASCVD, was responsible for almost 2.4 million fatalities in China in 2016, making it the primary cause of death globally [1]. One of the most significant risk factors is dyslipidemia [2, 3]. Medical literature has firmly established the correlation between ASCVD and low-density lipoprotein cholesterol (LDL-C) [4]. Large amounts of documents provided rigid proof that the LDL-C lowering effects of statins may decrease ASCVD occurrence over the last 30 years [5]. Even though statins and other treatments (e.g., cholesterol absorption inhibitors and PCSK9 inhibitors) decrease LDL-C, significant ASCVD residual risks continue [6–9]. An elevated risk has been reported to be associated with substances contained in triglyceride-rich lipoproteins (TGRLs), such as triglycerides (TGs) and/or cholesterol [10–13]. Although TG is recognized as a measure of cardiovascular risk, whether lowering TG levels improves cardiovascular outcomes has not been demonstrated [14–17]. Furthermore, hypertriglyceridemia (HTG, ≥ 5.6 mmol/L) is an independent predictor of acute pancreatitis. Controlling severe hypertriglyceridemia may prevent inflammation [18]. Globally, the prevalence of HTG ranges from 15 to 20% [19], with China having a frequency of 13.8% [20]. As a result, it seems that there is an unmet demand for HTG management.

Consuming higher amounts of omega-3 fatty acids, such as eicosapentaenoic acid (EPA) or docosahexaenoic acid (DHA), has been demonstrated to decrease the levels of total cholesterol [21]. These fatty acids may be found in oily fish [21]. Previous report announced that LDL-C levels may considerably increase in some patients with HTG, particularly those with extremely high TG levels, despite the fact that certain studies have shown that EPA or DHA has TG-lowering effects [22]. Recent ASCVD outcome studies have failed to demonstrate a cardiovascular (CV) advantage of omega-3 fatty acid combinations including both EPA and DHA [14, 23, 24]. On the other hand, it has been shown that purified EPA therapies are beneficial from a therapeutic perspective. The MARINE (Multi-center, plAcebo-controlled, Randomized, double-blINd, 12-week study with an open-label Extension) research was a trial using icosapent ethyl (Vascepa®) (IPE), a medicine that includes only EPA and no DHA, for TG-lowering [25]. This study also included an open-label extension for participants who completed the initial phase. According to its data, IPE was effective in lowering total TG levels free from an elevation in plasma concentration of LDL-C. Taking 4 g of IPE daily lowered the relative risk of initial ischemic events by 25% and diminished the relative risk of total events by 30% in statin-treated patients with persistently increased TG levels, according to the global reduction of cardiovascular events with IPE-intervention research (REDUCE-IT) [26, 27].

However, there is a scarcity of multi-center evidence on the consequences of IPE launched in a Chinese population-based cohort study. This study was aimed at assessing the safety and effectiveness of treating Chinese patients who have extremely high triglyceride levels (5.6 to 22.6 mmol/L).

## Materials and methods

### Study design and participants

Forty-nine Chinese institutions participated in this phase III clinical investigation. This research was carried out at many sites utilizing a randomized, placebo-controlled, double-blind trial design. The research followed the rules and guidelines set out by local and/or national independent ethics committees, as well as the principles outlined in the Helsinki Declaration. Institutional review boards authorized the protocol, consent forms, and other pertinent documentation. Before the trial began, all the patients signed informed consent forms. CTR20170362 was the registration number for this study.

Eligible participants were men and women over the age of 18 whose TG levels fell between 5.6–22.6 mmol/L (with a stable diet and physical activity). If statin medication (with or without ezetimibe) was used, the dosage must have been stable for at least four weeks before the TG qualifying visit (visit 3 [week 2]).

Participants were required to safely stop taking any medications before the screening visit and throughout the course of the study, including niacin doses greater than 200 mg daily, fish oil or other foods rich in omega-3 fatty acids, fibrates, and any herbal or food additives that may regulate lipid levels.

### Randomization

By using computerized data collection and minimum dynamic randomization, patients were segregated into three groups, with an equal distribution of patients in each group, resulting in a 1:1:1 ratio. According to the baseline plasma TG levels (less than or more than 8.5 mmol/L), gender, and whether or not they had previously taken a statin (yes or no), patients were allocated to one of the groups randomly.

### Procedures

**Fig. 1:**
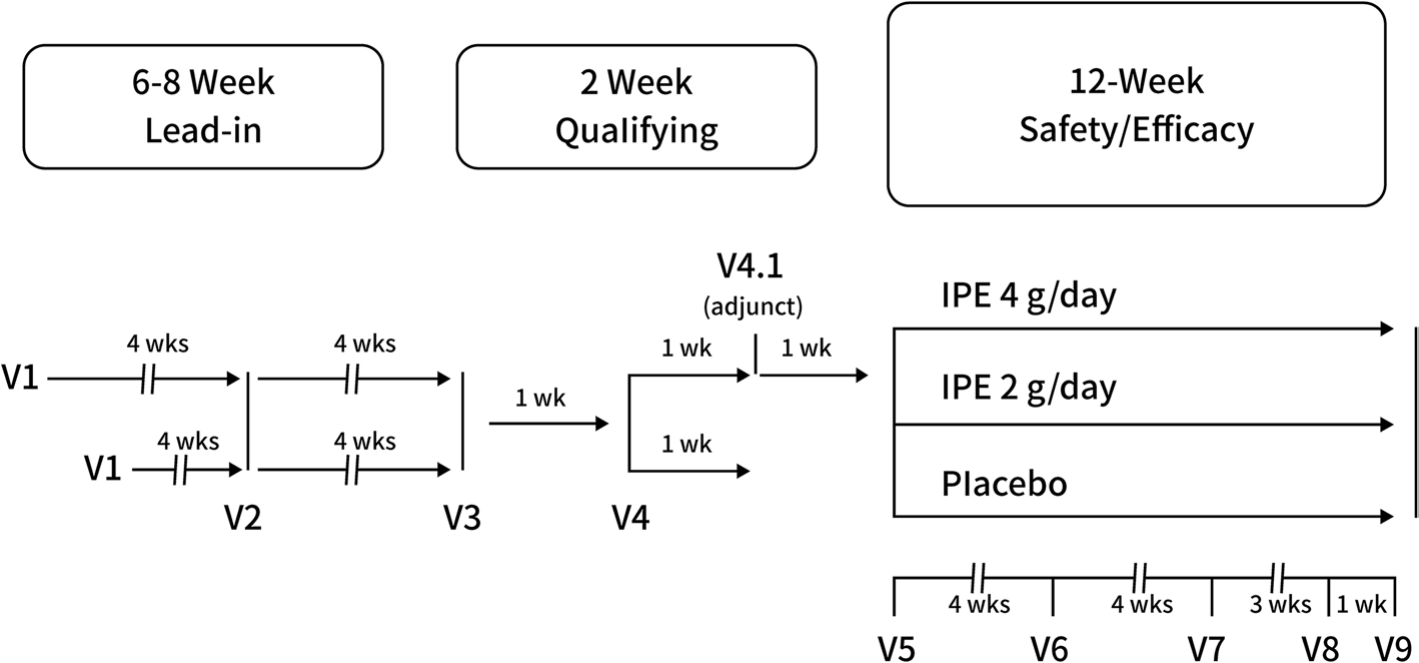
Study design. Abbreviation: IPE = icosapent ethyl

The framework of the study design was provided (Fig. 1). This clinical study consisted of a screening phase that lasted between 6 and 8 weeks, followed by a treatment phase that lasted 12 weeks and was double-blinded, random, and placebo-controlled. Patients who had a consistent diet, way of life, and medication regimen underwent a washout period after their first appointment.

For patients who had not received any other lipid-altering treatment and had been on a stable dosage of statin therapy for more than four weeks (with or without ezetimibe), this screening and stabilization phase lasted six weeks. For patients who stopped receiving other TG-altering treatments, it persisted for eight weeks (as mentioned above).

In order to ensure that the patients adhered to the lifestyle recommendations, visit 2 included a telephone interview. If patients' average overnight TG levels at visits 3 and 4 (separated by one week) were in the range of 5.6–22.6 mmol/L, they were eligible to participate in the following 12-week double-blind session. Patients were offered an optional TG measurement at visit 4.1 and an extra stabilization week if average fasting TG levels dropped outside of this range (one week later). According to the average fasting TG levels at visits 4 and 4.1, eligibility was reevaluated.

Those who met the inclusion criteria were monitored for a week before being randomly assigned to receive either 4 g/day of IPE (take two capsules of IPE, each with a dosage of 1 g, twice a day), 2 g/day of IPE (take one capsule of IPE with a dosage of 1 g and one capsule of placebo twice a day), or a placebo capsule (two placebo capsules twice daily) starting at visit 5 (week 0).

Mealtime, oral study medications were taken with or at the end of meals. All study medications (both IPE and placebo capsules) were provided by Amarin Pharmaceuticals Ireland Limited, which is based in Dublin, Ireland. Light liquid paraffin was used to fill each placebo capsule.

### Study outcomes

The median percentage difference in TG levels from the start to the end of the trial, after accounting for the placebo, was the major outcome measure (week 12). The TG baseline was determined using the mean TG value from visit 5 (week 0) as well as the value from the visit before that (one week earlier, visit 4 or visit 4.1). The mean values of TG at visits 8 (week 11) and 9 (visit 9) were used to compute the TG levels after the trial (week 12). The secondary objectives comprised median percentage changes in VLDL-C from baseline (visit 5, week 0) to the trial conclusion (visit 9, week 12). These adjustments were made to account for the placebo effects. The exploratory endpoints are low-density lipoprotein cholesterol (LDL-C), non-high-density lipoprotein cholesterol (non-HDL-C), and very low-density lipoprotein cholesterol (VLDL-TG).

### Safety assessments

Evaluations of safety were performed using information gathered from clinical laboratory tests, abdominal ultrasonography, vital signs, electrocardiograms, and physical examinations, as well as from reports of adverse events (AEs). AEs that appeared for the first time or were worse during the double-blind phase were known as treatment-emergent adverse events (TEAE).

### Statistical analysis

The primary objective was to determine the placebo-adjusted median percentage difference in fasting TG levels between weeks 0 and 12 of treatment. According to data from the MARINE trial, the difference in effectiveness between IPE 4 g/day and placebo was estimated to be 30% (51% standard deviation). With 100 patients in each group, IPE 4 g/day, and placebo, there would be 98.5% statistical test power at a two-sided significance level of 0.05 (based on the PASS 12.0 software). It was anticipated that there was a difference of 25.4% (with a standard deviation of 51%) between IPE 2 g/day and placebo (data from the MARINE study). If 100 patients were assigned to the IPE 2 g/day group, and 100 patients to the placebo group, the statistical test would have a power of 94% at a significance level of 0.05 for two-sided analysis (based on PASS 12.0 software). A total of 360 patients were intended for randomization, with 120 in each group, taking into account a 20% dropout rate from randomization until study completion.

The statistical evaluation was produced with SAS 9.4. For the main effectiveness analysis, the modified intent-to-treat (mITT) data set, per protocol set (PPS) data, and Safety Set (SS) data were used. A covariance model (ANCOVA) was used for the effectiveness analysis, with the treatment group, gender, and baseline statin usage as variables and baseline TG value as a covariate. If a normal distribution or homogeneity of variances could not be met, nonparametric intergroup comparisons were made using Wilcoxon rank-sum tests with the interquartile and median ranges for each group. Additionally, Hodges-Lehmann two-sided 95% and 99% CIs for the predicted median and treatment differences for each comparison across groups were supplied. In all two-sided statistical tests, the threshold for statistical significance was established at *P* less than 0.05 (unless otherwise stated).

## Results

### Patients

**Fig. 2:**
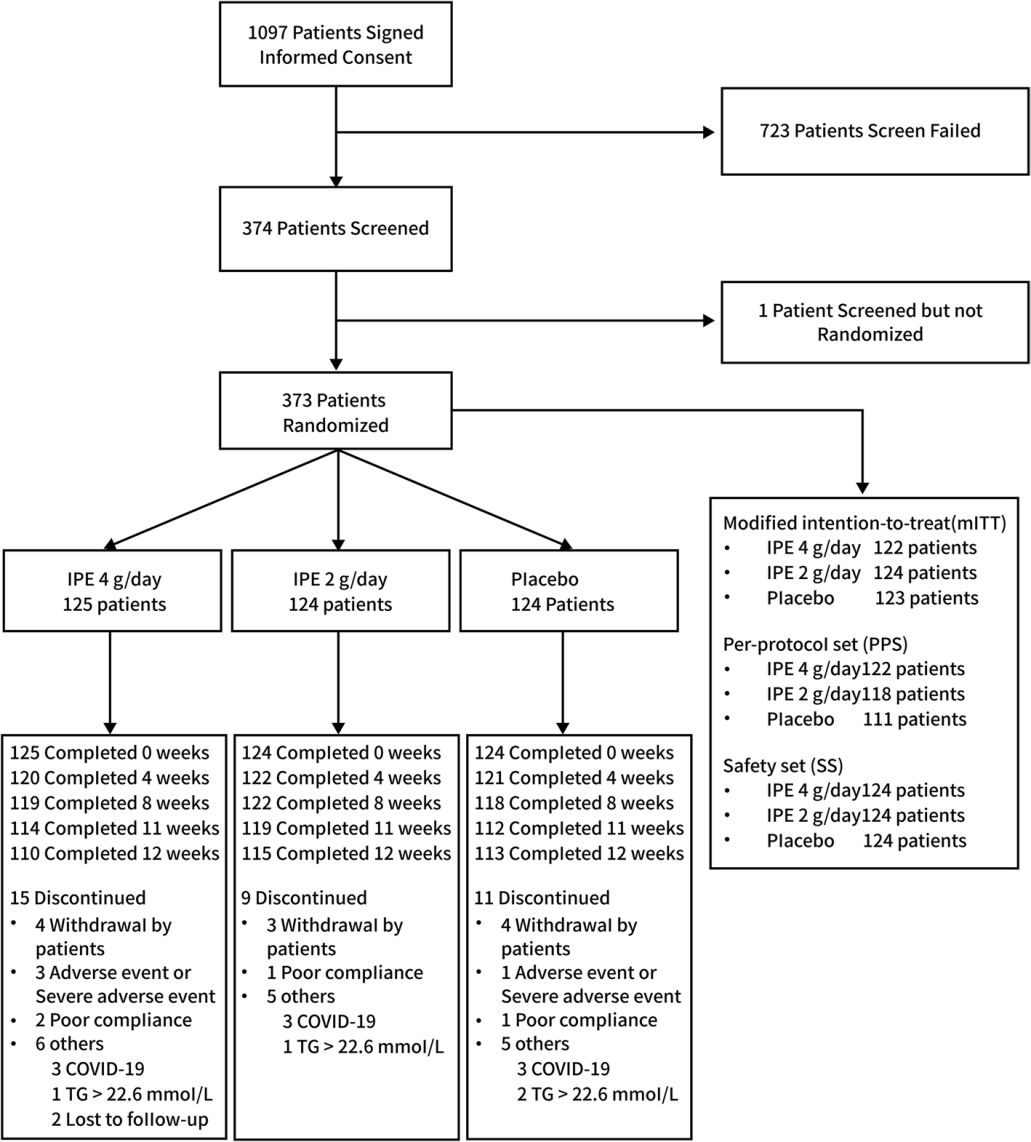
Patient disposition. Abbreviations: IPE = icosapent ethyl; TG = triglyceride; COVID-19 = coronavirus disease 2019

A total of 1097 patients gave their informed permission between December 2017 and April 2020. A total of 723 people failed the screening phase, while 374 patients finished it and underwent randomization. This 12-week experiment was successfully completed by 110 out of 125 patients (88.0%) in the group receiving 4 g IPE once a day, 115 out of 124 patients (92.7%) in the group receiving 2 g/day, and 113 out of 124 patients (91.1%) in the placebo group (Fig. 2). The list of patients' general demographic features (Table 1). The average age of patients was 48.9 years old. The mean body mass index was 26.6 kg/m^2^. The population was made up of 92.8% people of the Chinese Han ethnicity and 75.1% of men. 10.5% of the randomized mITT patients were taking statins, 20.6% had diabetes, and 48.3% had high blood pressure. 54.2% of the baseline TG levels were more than 8.5 mmol/L, with a median baseline TG level of 9.370 mmol/L. The median LDL-C level at baseline was 1.360 mmol/L. At baseline, patient characteristics were similar among the three therapy groups.

**Table 1:** Baseline characteristics

Characteristics	IPE 4 g/day (*n* = 125)	IPE 2 g/day (*n* = 124)	Placebo (*n* = 124)	Total (*n* = 373)
Age, mean ± SD (years)	48.5 ± 10.58	48.8 ± 10.44	49.3 ± 10.13	48.9 ± 10.36
Age ≤ 65 years, n (%)	118 (94.4%)	113 (91.1%)	116 (93.5%)	347 (93.0%)
Men, n (%)	93 (74.4%)	93 (75.0%)	94 (75.8%)	280 (75.1%)
Nationality, China (%)	125 (100.0%)	123 (99.2%)	124 (100.0%)	372 (99.7%)
Han ethnicity, n (%)	114 (91.2%)	116 (93.5%)	116 (93.5%)	346 (92.8%)
Height, mean ± SD (cm)	169.0 ± 7.76	169.1 ± 7.49	169.8 ± 7.26	169.3 ± 7.49
Weight, mean ± SD (kg)	76.5 ± 11.66	75.3 ± 11.91	78.4 ± 13.49	76.7 ± 12.41
Body mass index, mean ± SD (kg/m^2^)	26.7 ± 2.75	26.2 ± 3.03	27.1 ± 3.30	26.6 ± 3.05
Body mass index < 25, n (%)	33 (26.4%)	47 (37.9%)	33 (26.6%)	113 (30.3%)
Statin use	13 (10.4%)	13 (10.5%)	13 (10.5%)	39 (10.5%)
Baseline median TG (mmol/L)	9.470	9.215	9.270	9.370
Baseline TG > 8.5 mmol/L	67 (53.6%)	68 (54.8%)	67 (54.0%)	202 (54.2%)
Baseline median LDL-C (mmol/L)	1.420	1.290	1.360	1.360
Diabetes mellitus	23 (18.4%)	28 (22.6%)	26 (21.0%)	77 (20.6%)
Hypertension	56 (44.8%)	64 (51.6%)	60 (48.4%)	180 (48.3%)

All data were collected from the randomized population

*Abbreviations*: *TG* triglyceride, *LDL-C* low-density lipoprotein-cholesterol, *SD* standard deviation

### Efficacy endpoints

**Fig. 3:**
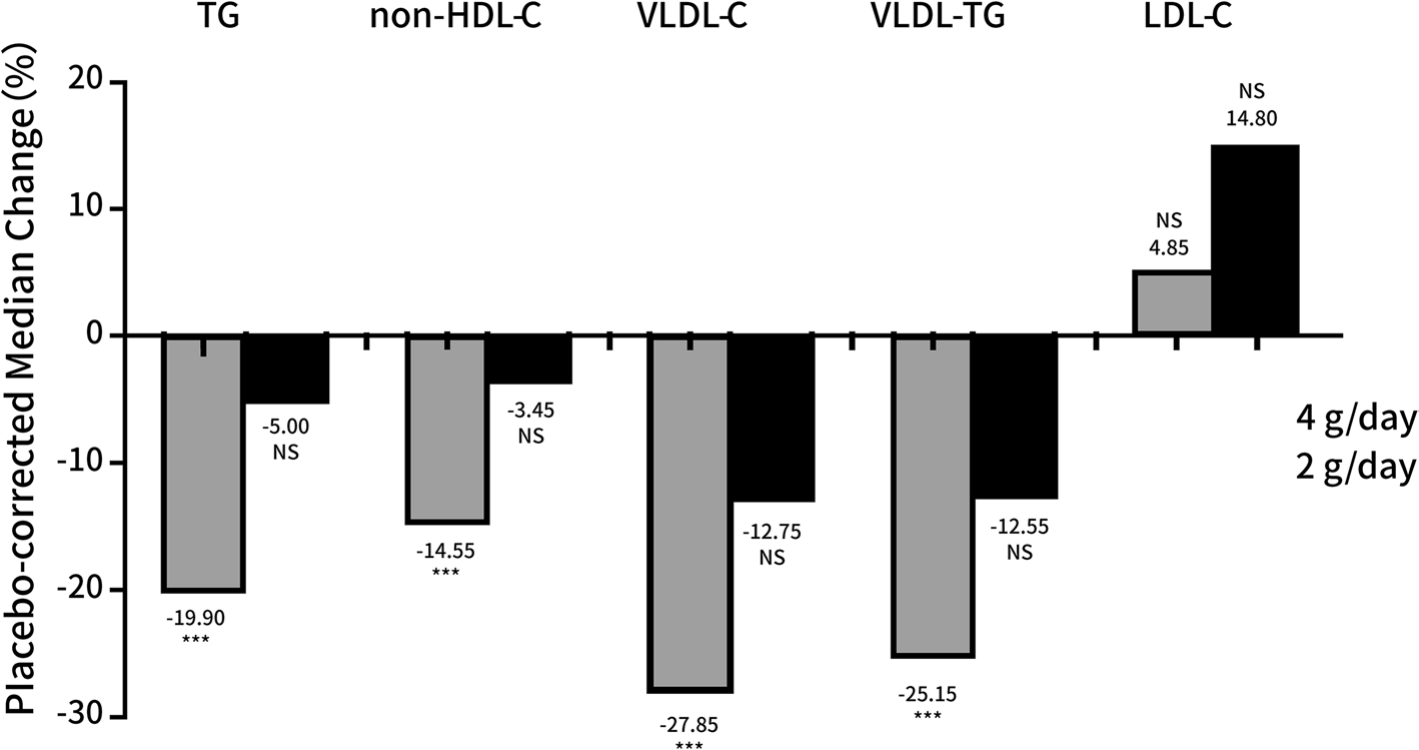
Changes in the median levels of lipids from the beginning of the study to the end of it in the modified intent-to-treat groups. Changes in lipid levels, after correcting for placebo, were shown in individuals with hypertriglyceridemia who took either IPE (4 g/day) or IPE (2 g/day) during the course of the study. ***P < 0.001. NS indicates that the difference between the groups is not statistically significant

The findings obtained for the main effectiveness objectives were shown in Fig. 3 and Table 2. According to the analyses from the mITT trial population, blood TG levels were reduced by 28.4%, 12.0%, and 6.2% in the groups that received placebo, IPE 2 g/day, and IPE 4 g/day, respectively, compared to baseline values. It was shown that IPE at a dose of 4 g per day reduced total cholesterol levels by 19.9% relative to baseline (95% confidence interval [CI]: 29.8% to 10.0%, *P* < 0.001). However, IPE at a dosage of 2 g per day led a TG level decrease by only 5.0% (95% CI: 16.2% to 6.2%, *P* = 0.361). There was a trend toward decreased TG levels from baseline, but no differences with statistical significance was observed between the IPE 2 g/day and placebo groups. The findings from the PPS and the mITT population were comparable. The primary endpoint of the mITT population was analyzed in subgroups (Table 2). The findings of the subgroup analysis (based on stratification variables of baseline TG levels, gender, and statin usage) were identical to those of the whole research, with IPE 4 g/day considerably lowering TG levels in comparison to placebo. Figure 3 and Table 3 show the secondary and exploratory effectiveness endpoints respectively. Other TG-related lipid markers were considerably reduced by 4 g of IPE per day, including non-HDL-C by 14.6%, VLDL-C by 27.9%, and distribution or homogeneity by 25%. Neither IPE 4 g/day nor 2 g/day elevated LDL-C levels considerably compared to placebo (Table 3).

**Table 2:** Primary endpoint: alteration in triglyceride (TG) levels from baseline to week 12 (modified intent-to-treat population)

Characteristics	IPE 4 g/day (*n* = 125)			IPE 2 g/day (*n* = 124)			Placebo (*n* = 124)			Placebo-corrected median TG Change from baseline	
	Baseline TG (mmol/L)	End-of-treatment TG (mmol/L)	Change from baseline (%)	Baseline TG (mmol/L)	End-of-treatment TG (mmol/L)	Change from baseline (%)	Baseline TG (mmol/L)	End-of-treatment TG (mmol/L)	Change from baseline (%)	IPE 4 g/day vs. placebo (%, ***P*** value)	IPE 2 g/day vs. placebo (%, ***P*** value)
Intent-to-treat population (*n* = 122, 124, 123)	9.455 (5.910)	6.145 (5.370)	− 28.35 (56.40)	9.215 (6.410)	7.720 (7.055)	− 11.95 (63.85)	9.170 (5.580)	8.290 (6.070)	− 6.20 (67.40)	− 19.90, < 0.001	− 5.00, 0.361
Baseline TG ≤ 8.5 mmol/L (*n* = 58, 56, 57)	7.145 (3.380)	5.320 (2.170)	− 29.40 (40.90)	6.900 (3.165)	6.010 (4.150)	− 1.200 (59.45)	6.860 (3.290)	6.760 (4.020)	0.50 (58.00)	− 25.95, < 0.001	− 5.90, 0.570
Baseline T > 8.5 mmol/L (*n* = 64, 68, 66)	11.715 (5.870)	8.700 (6.550)	− 27.60 (64.80)	11.430 (5.725)	9.625 (6.675)	− 17.70 (66.45)	11.415 (4.610)	10.505 (7.190)	− 17.80 (77.00)	− 15.30, 0.030	− 4.75, 0.509
Male (*n* = 90, 93, 94)	9.575 (5.710)	6.340 (5.880)	− 27.15 (56.40)	9.090 (5.470)	7.240 (7.010)	− 8.40 (65.60)	9.065 (4.980)	8.780 (5.710)	− 5.00 (69.60)	− 19.95, 0.002	− 4.65, 0.511
Female (*n* = 32, 31, 29)	7.965 (5.635)	4.820 (4.120)	− 34.70 (53.20)	10.860 (7.260)	8.300 (6.410)	− 13.20 (44.20)	9.740 (6.510)	6.970 (6.510)	− 13.30 (62.10)	− 20.35, 0.034	− 7.75, 0.506
Statin use at baseline (*n* = 13, 13, 13)	8.610 (5.480)	6.460 (4.600)	− 13.10 (37.20)	13.720 (7.240)	9.360 (8.430)	− 9.20 (75.50)	9.170 (6.250)	10.390 (4.020)	− 17.70 (66.70)	− 9.15, 0.739	− 8.20, 0.505
No statin use at baseline (*n* = 109, 111, 110)	9.470 (5.740)	6.120 (5.370)	− 29.40 (59.10)	9.010 (5.690)	7.220 (7.050)	− 12.00 (64.10)	9.245 (5.080)	8.075 (6.130)	− 5.85 (66.10)	− 21.45, < 0.001	− 4.60, 0.439

Data are shown as the median (interquartile range) for endpoint values

*Abbreviation*: *TG* triglyceride

**Table 3:** Secondary and exploratory efficacy endpoints (modified intent-to-treat population)

Characteristics	IPE 4 g/day (*n* = 125)			IPE 2 g/day (*n* = 124)			Placebo (*n* = 124)			Placebo-corrected median change from baseline	
	Baseline Value	End-of-treatment Value	Change from baseline (%)	Baseline Value	End-of-treatment Value	Change from baseline (%)	Baseline Value	End-of-treatment Value	Change from baseline (%)	IPE 4 g/day vs. placebo (%, ***P*** Value)	IPE 2 g/day vs. placebo (%, ***P*** value)
VLDL-C (mmol/L) (*n* = 121, 124, 123)	2.850 (1.610)	2.170 (1.560)	− 17.20 (62.00)	2.750 (1.985)	2.875 (2.345)	− 5.50 (77.60)	3.030 (1.660)	3.290 (2.290)	9.60 (65.00)	− 27.85, < 0.001	− 12.75, 0.059
LDL-C (mmol/L) (*n* = 121, 124, 123)	1.430 (1.170)	1.650 (1.360)	11.80 (88.60)	1.290 (1.170)	1.755 (1.625)	25.20 (93.60)	1.350 (1.050)	1.460 (1.150)	3.80 (71.50)	4.85, 0.475	14.80, 0.065
Non–HDL-C (mmol/L) (*n* = 121, 124, 123)	4.690 (1.440)	4.260 (1.410)	− 6.60 (32.50)	4.785 (1.900)	4.900 (2.050)	5.25 (32.55)	4.690 (1.490)	5.020 (1.860)	8.80 (33.50)	− 14.55, < 0.001	− 3.45, 0.284
VLDL-TG (mmol/L) (*n* = 121, 123,123)	6.870 (5.880)	4.630 (4.510)	− 25.50 (63.70)	6.890 (6.920)	6.300 (7.380)	− 12.00 (84.60)	6.720 (5.680)	6.760 (6.920)	3.20 (83.80)	− 25.15, < 0.001	− 12.55, 0.114

Data are shown as the median (interquartile range) for endpoint values

*Abbreviations*: *TG* triglyceride, *non-HDL-C* non-high-density lipoprotein cholesterol, *VLDL-C* very low-density lipoprotein cholesterol, *LDL-C* low-density lipoprotein cholesterol, *VLDL-TG* very low-density lipoprotein-triglycerides

### Safety

In all therapy groups, the occurrence of TEAE was typically modest and comparable. Most TEAEs were modest and were unrelated to study medications. Drug-related TEAEs with a greater incidence (> 2%) included diarrhea (9.7%, 2.4%, and 5.6% in IPE 2 g/day, 4 g/day and placebo, respectively). Serious TEAEs were recorded with a low incidence (2.4%, 3.2%, 2.4% in IPE 2 g/day, 4 g/day and placebo, respectively). There were no drug-related severe TEAEs. Due to TEAEs, three patients in the IPE 4 g/day group (2.4%; one patient each with abnormal hepatic function, urticaria, and severe pancreatitis) and one patient in the placebo group (0.8%; hypersensitive response) terminated the trial. Only two incidences of drug-related TEAEs resulting in the withdrawal of the research (one each in IPE 4 g/day was urticaria and in the placebo group was a hypersensitive response) was detected. Glucose and HbA1c responses were comparable across the IPE and placebo groups. Vital signs, electrocardiography, liver function tests, and serum creatinine phosphokinase levels were not statically significant after different IPE dosing treatments.

## Discussion

Triglyceride-rich lipoproteins (TGRLs) are closely associated with the pathogenesis and progression of atherosclerotic cardiovascular disease (ASCVD) via direct arterial wall deposition, endothelial dysfunction, recruitment of monocytes and subsequent foam cell formation, stimulation of inflammatory cytokines, and activation of platelets [28]. The precise mechanism by which HTG causes acute pancreatitis is unknown. This could be due to the catalysis of TG by pancreatic lipase into free fatty acids (FFA), which induces ischemia and injury to pancreatic cells [18]. According to strengrecent research, those with TG levels > 5.6 mmol/L are 1.79 times more likely to have pancreatitis episodes than individuals who have TG levels under 5.6 mmol/L [29]. Acute pancreatitis is a medical emergency that may be avoided by proactive VHTG management, which may improve clinical and economic results [29].

IPE (Vascepa®) is an omega-3 fatty acid medication possessing a highly refined (96%) EPA ethyl ester that is devoid of DHA [25]. It is marketed under the brand name Vascepa. Because of its one-of-a-kind effects on synthesis of VLDL-C, metabolism of TG in hepatic cells, and removal of lipoprotein, in addition to its anti-inflammatory, antiplatelet, and antithrombotic actions [25, 26], it is able to suppress the processes that lead to atherosclerosis. This is the first large-scale, multi-center, phase III study in a Chinese population to examine the TG-lowering effects of IPE in patients having extremely high TG concentrations. The goal of this research was to determine whether or not IPE lowered TG levels.

For the main effectiveness objective, IPE at 4 g per day lowered TG levels by 28.4% from baseline and substantially decreased TG levels by 19.9% when adjusted for a placebo. Difference in terms of TG level changes between IPE 2 g/day and placebo didn’t reach the significance level, but there was a numerical decrease in TG levels (median 12.0%) from baseline. At 4 g/day, IPE substantially lowered VLDL-C levels by a median of 27.9% when adjusted for placebo and also decreased other TG-related lipid profiles (non-HDL-C and VLDL-TG). At 2 g per day, IPE also exhibited reducing effects, but this was not statistically significant ones. These findings showed that IPE has a dose-dependent impact, with a maximum benefit of 4 g per day.

Moreover, compared to placebo, IPE did not substantially elevate LDL-C levels in this investigation, as is often reported with mixed omega-3 fatty acid agents (including both EPA and DHA) in comparable patient populations [30, 31].

While the magnitude of the median placebo-adjusted TG reduction in the current study appears to be slightly lower than that in the MARINE study (-5.0% and -19.9% at 2 g/day, and -19.9% and -33.1% at 4 g/day, respectively), the within-group percent differences from baseline to 12-week endpoint in each IPE group are very similar to the changes in the MARINE study (12.0% vs 19.9% in 2 g/day, and 28.4% vs 33.1% in 4 g/day, respectively). The following are possible explanations for the apparent disparities between these two investigations. To begin, the baseline TG level differed between the two trials. According to the findings of this research, the TG level at baseline was higher than what was seen in the MARINE study (median 9.37 mmol/L vs 7.67 mmol/L, and the proportion of TG > 8.5 mmol/L is 54.2% vs 39.3%, respectively). The greater the TG level, the more difficult it is to lower it. Second, in this trial, TG fell by a median of 6.2% from baseline in the placebo group, but increased by a median of 9.7% in the MARINE placebo group. This might be attributed to improved nutrition management in the Chinese research population. As a result, in the analysis, the placebo-corrected decrease seemed to have been limited. IPE at 4 g/day demonstrated comparable TG-lowering effects in subgroup analyses with varying baseline TG levels, sex, and statin usage to those of the whole mITT sample. As a result, IPE effects were predictable across patient subgroups.

The MARINE study showed that treatment with 4 g or 2 g /day of IPE for 12 weeks statistically significantly increased EPA concentration (in both plasma and red blood cells) in a dose-dependent manner compared to placebo. A linear relationship between EPA levels (in plasma and red blood cells) and TG reduction was observed. Patients in the IPE 4 g/day group had a larger mean percent increase in EPA concentration (in plasma and in red blood cells) and a larger median percent decrease in TG from baseline than patients in the IPE 2 g/day group and the placebo group. Mean levels of plasma and red blood cell fatty acid parameters EPA and DPAn-3 (a metabolite of EPA) also increased in a dose-dependent manner.

The results of China phase I study and the population pharmacokinetic (PopPK) analysis of IPE phase I studies in China and the United States showed that within the dose range of 2 g/day to 4 g/day, the baseline-corrected plasma total EPA, red blood cell total EPA and plasma unesterified EPA concentration showed dose proportional property. The dose effect of IPE 2 g/day and 4 g/day was linear. There was no significant difference in the plasma EPA exposure level between Chinese and Americans. It indicates that PD/PK study of IPE has consistency, and the TG lowering effect in different ethnic populations are consistent, and there is no significant racial difference.

IPE has been commercially used around the world for many years. Piles of clinical data and experience has been accumulated. Based on the results of phase I studies in China and abroad, it showed the linear correlation between IPE, EPA and TG. The phase III results are consistent with PD/PK studies, which predict that there should be a linear relationship between EPA levels (in plasma and red blood cells) and TG reduction after IPE treatment in the Chinese population. And the main purpose of the phase III study in China is to evaluate the effectiveness and safety of reducing TG after 12 weeks of IPE treatment in the Chinese population. At the same time, TG is a routine clinical detection item in China, and patients generally do not detect EPA in Chinese clinical practice. Therefore, this study set the primary endpoint as TG and did not measure EPA. EPA levels were also examined in the REDUCE-IT study, and higher EPA levels were found to confer cardiovascular benefits. The time of this study was short, and the study endpoints did not involve long-term follow-up and reduction of cardiovascular events, which is one of the reasons why this study did not design to detect EPA.

Since this study lacking in the detection of EPA level, larger sample studies will continue to carry out in the Chinese population based on the above research data, and further explore the relationship between EPA levels and TG-lowering efficacy and cardiovascular risk reduction. Unfortunately, this study currently unable to additionally detect the level of EPA because of the original study design and locked database.The acceptability and safety profile results of IPE were consistent with those of prior research [25–27, 32, 33]. IPE was generally well tolerated in all investigations, including Chinese individuals, with a TEAE incidence and severity rate comparable to those of placebo.

Apart from these, other polyunsaturated fatty acids have also been shown to process lipid-modifying effects, such as linolenic acid and dihomo-γ-linolenic acid (DGLA). It is well established LA consumption leads to lower cholesterol level [34]. A recent analysis pooled results from a series of prospective cohort studies showed that higher LA intake was associated with a modestly lower risk of mortality from all causes, such as cardiovascular disease and cancer [35]. Observational data reveals that low circulating DGLA level is associated with total mortality in patients with acute cardiovascular disease and acute decompensated heart failure [36], diabetic retinopathy [37]; while A high serum DGLA level was associated with obesity, body fat accumulation, a high ALT level, and insulin resistance in patients with type 2 diabetes [38]. However, the TG-lowering effect of other fatty acids is still in the exploratory stage. According to clinical guidelines and clinical evidence, omega-3 polyunsaturated fatty acids (IPE) is more recommended for lipid lowering. In the future, more basic or large-scale research data may be needed to explore the effects of other fatty acids in reducing TG and reducing cardiovascular risk, as well as their differences in efficacy. In this study, IPE was used, which is a kind of pure EPA preparation, and the effect of other fatty acids on TG could not be assessed.

### Comparisons with other studies and what does the current work add to the existing knowledge

According to previously published analyses in a non-Chinese population, the MARINE study demonstrated that 4 g/day IPE reduced within-group TG concentrations by 26.6% in patients with extremely high TG levels (5.6—22.6 mmol/L) without significant increases in LDL-C levels compared to placebo. Our results were consistent with the findings of MARINE study [25].

Regarding the clinical advantages on cardio-cerebrovascular disease, it was determined that a daily dosage of 4 g of IPE reduced the risk of nonfatal myocardial infarction, cardiovascular mortality, coronary angioplasty, nonfatal stroke, or unstable angina by 25% [27], and the risk of total events by 30% [26] in patients whose LDL-C levels were under control but whose TG levels were elevated. These were the findings of the REDUCE-IT study [26, 27]. After the publication of the REDUCE-IT report, a number of worldwide treatment recommendations [39–41] began recommending IPE as an additional therapy option. The authors believe that the REDUCE-IT study's findings might be used to further support the therapeutic benefits of cardiovascular risk reduction in Chinese patients, because the findings from the Phase III trial in China that were published in this article are comparable to the findings of the MARINE research. MARINE was conducted in the same country as that in this study. Additional clinical studies on appropriate patient populations are also necessary.

### Strengths and limitations

This work included comprehensive analyses of data in Chinese patients, which allowed for a more targeted evaluation of IPE's effectiveness and safety in this population. These data indicated that IPE was effective for the treatment of very high triglyceride condition in Chinese patients, and that it was well-tolerated. Furthermore, the study was able to identify an effective dosage for Chinese individuals, which may be different from the dosage used in other Asian populations. However, this study did not measure EPA levels or its metabolites. This is a limitation of the current work, which requires future work to explore the relationship between EPA levels and TG-lowering efficacy and cardiovascular risk reduction. The time of this study was short, and the study endpoints did not involve long-term follow-up and reduction of cardiovascular events, which is another limitation of this work. In addition, this study did not set up a comparison with other fatty acids, so there is still a lack of difference in the efficacy of EPA in reducing plasma TG level compared with other fatty acids. The cardiovascular benefits of EPA or other fatty acids may need to be further consolidated in a new randomized controlled study. In the future, more evidence is needed to further explore and compare the mechanism and efficacy of various fatty acids.

## Conclusion

In the Chinese population, IPE at 4 g/day could efficiently and safely diminish serum TG by 19.9% when compared to placebo, 28.4% when compared to baseline, and also reduce other critical TG-related lipids in individuals with extremely high TG levels (5.6–22.3 mmol/L) without significantly increasing LDL-C. Both doses of IPE were well tolerated, but the optimal benefit was obtained from the 4 g/day dose, which also had a tolerability that was equivalent to that of a placebo. The current findings validated the effectiveness and safety of IPE in a Chinese patient group, consistent with the findings of the MARINE trial in the Western community.

## Data Availability

The entirety of the data featured in this work is made accessible to interested parties through the author of correspondence.

## References

[1] Pasquel FJ, Gregg EW, Ali MK (2018). The evolving epidemiology of atherosclerotic cardiovascular disease in people with diabetes. Endocrinol Metab Clin North Am.

[2] Libby P, Buring JE, Badimon L, Hansson GK, Deanfield J, Bittencourt MS, Tokgozoglu L, Lewis EF (2019). Atherosclerosis. Nat Rev Dis Primers.

[3] Ryan A, Heath S, Cook P (2018). Dyslipidaemia and cardiovascular risk. BMJ.

[4] Ference BA, Ginsberg HN, Graham I, Ray KK, Packard CJ, Bruckert E, Hegele RA, Krauss RM, Raal FJ, Schunkert H (2017). Low-density lipoproteins cause atherosclerotic cardiovascular disease. 1. Evidence from genetic, epidemiologic, and clinical studies. A consensus statement from the European Atherosclerosis Society Consensus Panel. Eur Heart J.

[5] Silverman MG, Ference BA, Im K, Wiviott SD, Giugliano RP, Grundy SM, Braunwald E, Sabatine MS (2016). Association between lowering LDL-C and cardiovascular risk reduction among different therapeutic interventions: a systematic review and meta-analysis. JAMA.

[6] Schwartz GG, Steg PG, Szarek M, Bhatt DL, Bittner VA, Diaz R, Edelberg JM, Goodman SG, Hanotin C, Harrington RA (2018). Alirocumab and cardiovascular outcomes after acute coronary syndrome. N Engl J Med.

[7] Sabatine MS, Giugliano RP, Keech AC, Honarpour N, Wiviott SD, Murphy SA, Kuder JF, Wang H, Liu T, Wasserman SM (2017). Evolocumab and clinical outcomes in patients with cardiovascular disease. N Engl J Med.

[8] Cannon CP, Blazing MA, Giugliano RP, McCagg A, White JA, Theroux P, Darius H, Lewis BS, Ophuis TO, Jukema JW (2015). Ezetimibe added to statin therapy after acute coronary syndromes. N Engl J Med.

[9] Sampson UK, Fazio S, Linton MF (2012). Residual cardiovascular risk despite optimal LDL cholesterol reduction with statins: the evidence, etiology, and therapeutic challenges. Curr Atheroscler Rep.

[10] Chait A, Ginsberg HN, Vaisar T, Heinecke JW, Goldberg IJ, Bornfeldt KE (2020). Remnants of the triglyceride-rich lipoproteins, diabetes, and cardiovascular disease. Diabetes.

[11] Marston NA, Giugliano RP, Im K, Silverman MG, O'Donoghue ML, Wiviott SD, Ference BA, Sabatine MS (2019). Association between triglyceride lowering and reduction of cardiovascular risk across multiple lipid-lowering therapeutic classes: a systematic review and meta-regression analysis of randomized controlled trials. Circulation.

[12] Mach F, Baigent C, Catapano AL, Koskinas KC, Casula M, Badimon L, Chapman MJ, De Backer GG, Delgado V, Ference BA (2020). 2019 ESC/EAS Guidelines for the management of dyslipidaemias: lipid modification to reduce cardiovascular risk. Eur Heart J.

[13] Nordestgaard BG (2016). Triglyceride-rich lipoproteins and atherosclerotic cardiovascular disease: new insights from epidemiology, genetics, and biology. Circ Res.

[14] Nicholls SJ, Lincoff AM, Garcia M, Bash D, Ballantyne CM, Barter PJ, Davidson MH, Kastelein JJP, Koenig W, McGuire DK (2020). Effect of high-dose omega-3 fatty acids vs corn oil on major adverse cardiovascular events in patients at high cardiovascular risk: the STRENGTH randomized clinical trial. JAMA.

[15] Landray MJ, Haynes R, Hopewell JC, Parish S, Aung T, Tomson J, Wallendszus K, Craig M, Jiang L, Group HTC (2014). Effects of extended-release niacin with laropiprant in high-risk patients. N Engl J Med.

[16] Investigators A-H, Boden WE, Probstfield JL, Anderson T, Chaitman BR, Desvignes-Nickens P, Koprowicz K, McBride R, Teo K, Weintraub W (2011). Niacin in patients with low HDL cholesterol levels receiving intensive statin therapy. N Engl J Med.

[17] Ginsberg HN, Elam MB, Lovato LC, Crouse JR, Leiter LA, Linz P, Friedewald WT, Buse JB, Gerstein HC, Group AS (2010). Effects of combination lipid therapy in type 2 diabetes mellitus. N Engl J Med.

[18] Garg R, Rustagi T (2018). Management of hypertriglyceridemia induced acute pancreatitis. Biomed Res Int.

[19] Parhofer KG, Laufs U (2019). The diagnosis and treatment of hypertriglyceridemia. Dtsch Arztebl Int.

[20] Zhang M, Deng Q, Wang L, Huang Z, Zhou M, Li Y, Zhao Z, Zhang Y, Wang L (2018). Prevalence of dyslipidemia and achievement of low-density lipoprotein cholesterol targets in Chinese adults: a nationally representative survey of 163,641 adults. Int J Cardiol.

[21] Skulas-Ray AC, Wilson PWF, Harris WS, Brinton EA, Kris-Etherton PM, Richter CK, Jacobson TA, Engler MB, Miller M, Robinson JG (2019). Omega-3 fatty acids for the management of hypertriglyceridemia: a science advisory from the American Heart Association. Circulation.

[22] Innes JK, Calder PC (2018). The differential effects of eicosapentaenoic acid and docosahexaenoic acid on cardiometabolic risk factors: a systematic review. Int J Mol Sci.

[23] Manson JE, Cook NR, Lee IM, Christen W, Bassuk SS, Mora S, Gibson H, Albert CM, Gordon D, Copeland T (2019). Marine n-3 fatty acids and prevention of cardiovascular disease and cancer. N Engl J Med.

[24] Bowman L, Mafham M, Wallendszus K, Stevens W, Buck G, Barton J, Murphy K, Aung T, Haynes R, Group ASC (2018). Effects of n-3 fatty acid supplements in diabetes mellitus. N Engl J Med.

[25] Bays HE, Ballantyne CM, Kastelein JJ, Isaacsohn JL, Braeckman RA, Soni PN (2011). Eicosapentaenoic acid ethyl ester (AMR101) therapy in patients with very high triglyceride levels (from the Multi-center, plAcebo-controlled, Randomized, double-blINd, 12-week study with an open-label Extension [MARINE] trial). Am J Cardiol.

[26] Bhatt DL, Steg PG, Miller M, Brinton EA, Jacobson TA, Ketchum SB, Doyle RT, Juliano RA, Jiao L, Granowitz C (2019). Effects of icosapent ethyl on total ischemic events: from REDUCE-IT. J Am Coll Cardiol.

[27] Bhatt DL, Steg PG, Miller M, Brinton EA, Jacobson TA, Ketchum SB, Doyle RT, Juliano RA, Jiao L, Granowitz C (2019). Cardiovascular risk reduction with icosapent ethyl for hypertriglyceridemia. N Engl J Med.

[28] Peng J, Luo F, Ruan G, Peng R, Li X (2017). Hypertriglyceridemia and atherosclerosis. Lipids Health Dis.

[29] Christian JB, Arondekar B, Buysman EK, Johnson SL, Seeger JD, Jacobson TA (2012). Clinical and economic benefits observed when follow-up triglyceride levels are less than 500 mg/dL in patients with severe hypertriglyceridemia. J Clin Lipidol.

[30] Kastelein JJ, Maki KC, Susekov A, Ezhov M, Nordestgaard BG, Machielse BN, Kling D, Davidson MH (2014). Omega-3 free fatty acids for the treatment of severe hypertriglyceridemia: the EpanoVa fOr Lowering Very high triglyceridEs (EVOLVE) trial. J Clin Lipidol.

[31] Harris WS, Ginsberg HN, Arunakul N, Shachter NS, Windsor SL, Adams M, Berglund L, Osmundsen K (1997). Safety and efficacy of Omacor in severe hypertriglyceridemia. J Cardiovasc Risk.

[32] Bhatt DL, Steg PG, Miller M, Brinton EA, Jacobson TA, Jiao L, Tardif JC, Gregson J, Pocock SJ, Ballantyne CM, Investigators R-I (2019). Reduction in first and total ischemic events with icosapent ethyl across baseline triglyceride tertiles. J Am Coll Cardiol.

[33] Ballantyne CM, Bays HE, Kastelein JJ, Stein E, Isaacsohn JL, Braeckman RA, Soni PN (2012). Efficacy and safety of eicosapentaenoic acid ethyl ester (AMR101) therapy in statin-treated patients with persistent high triglycerides (from the ANCHOR study). Am J Cardiol.

[34] Ramsden CE, Zamora D, Majchrzak-Hong S, Faurot KR, Broste SK, Frantz RP, Davis JM, Ringel A, Suchindran CM, Hibbeln JR (2016). Re-evaluation of the traditional diet-heart hypothesis: analysis of recovered data from Minnesota Coronary Experiment (1968–73). BMJ.

[35] Li J, Guasch-Ferré M, Li Y, Hu FB (2020). Dietary intake and biomarkers of linoleic acid and mortality: systematic review and meta-analysis of prospective cohort studies. Am J Clin Nutr.

[36] Ouchi S, Miyazaki T, Shimada K, Sugita Y, Shimizu M, Murata A, Kato T, Aikawa T, Suda S, Shiozawa T (2017). Decreased circulating dihomo-gamma-linolenic acid levels are associated with total mortality in patients with acute cardiovascular disease and acute decompensated heart failure. Lipids Health Dis.

[37] Okamura T, Nakajima H, Hashimoto Y, Majima S, Senmaru T, Ushigome E, Nakanishi N, Hamaguchi M, Asano M, Yamazaki M (2021). Low circulating dihomo-gamma-linolenic acid is associated with diabetic retinopathy: a cross sectional study of KAMOGAWA-DM cohort study. Endocr J.

[38] Tsurutani Y, Inoue K, Sugisawa C, Saito J, Omura M, Nishikawa T (2018). Increased serum dihomo-γ-linolenic acid levels are associated with obesity, body fat accumulation, and insulin resistance in Japanese patients with type 2 diabetes. Intern Med.

[39] American Diabetes A (2021). 10. Cardiovascular disease and risk management: standards of medical care in diabetes-2021. Diabetes Care.

[40] Jia X, Al Rifai M, Hussain A, Martin S, Agarwala A, Virani SS (2020). Highlights from Studies in Cardiovascular Disease Prevention Presented at the Digital 2020 European Society of Cardiology Congress: Prevention Is Alive and Well. Curr Atheroscler Rep.

[41] Orringer CE, Jacobson TA, Maki KC (2019). National Lipid Association Scientific Statement on the use of icosapent ethyl in statin-treated patients with elevated triglycerides and high or very-high ASCVD risk. J Clin Lipidol.

